# Observation of sagittal X-ray diffraction by surface acoustic waves in Bragg geometry^1^

**DOI:** 10.1107/S1600576717002977

**Published:** 2017-03-14

**Authors:** Simone Vadilonga, Ivo Zizak, Dmitry Roshchupkin, Emelin Evgenii, Andrei Petsiuk, Wolfram Leitenberger, Alexei Erko

**Affiliations:** a Helmholtz Zentrum Berlin für Materialien und Energie, Germany; b Freie Universität Berlin, Germany; c Institute of Microelectronics Technology and High Purity Materials, Moscow, Russian Federation; d Universität Potsdam, Potsdam – Golm, Germany

**Keywords:** surface acoustic waves, optics, synchrotron radiation, sagittal X-ray diffraction

## Abstract

X-ray Bragg diffraction in sagittal geometry on a Y-cut langasite crystal (La_3_Ga_5_SiO_14_) modulated by Λ = 3 µm Rayleigh surface acoustic waves was studied at the BESSY II synchrotron radiation facility. Owing to the crystal lattice modulation by the surface acoustic wave diffraction, satellites appear. Their intensity and angular separation depend on the amplitude and wavelength of the ultrasonic superlattice. Experimental results are compared with the corresponding theoretical model that exploits the kinematical diffraction theory. This experiment shows that the propagation of the surface acoustic waves creates a dynamical diffraction grating on the crystal surface, and this can be used for space–time modulation of an X-ray beam.

## Abstract 2

X-ray Bragg diffraction in sagittal geometry on a Y-cut langasite crystal (La_3_Ga_5_SiO_14_) modulated by Λ = 3 µm Rayleigh surface acoustic waves was studied at the BESSY II synchrotron radiation facility. The surface acoustic waves create a dynamical diffraction grating on the crystal surface, which can be used for space–time modulation of an X-ray beam.

## 1. Introduction   

The interaction between crystalline materials and acoustic waves has been receiving a lot of interest recently. Acoustic wave properties, especially the wavelength and phase velocity, are strongly influenced by very subtle changes in the crystal and its environment. In a piezoelectric material, acoustic waves are coupled to electrical oscillations which can be easily controlled and measured. Additionally, since the deformation induced by acoustic waves in the crystal is periodic, it can be studied by diffraction experiments. This interaction can also be used to control the X-rays electrically and to introduce electrically controlled optical elements, reducing the necessity of mechanical motion in adaptive optics. This represents an interesting development for the optics for third- and fourth-generation UV and X-ray light sources. There have been different approaches in recent times to investigating the interaction between photons and different kinds of acoustic waves. The deformation induced in the crystal lattice by bulk acoustic waves has been studied (Blagov *et al.*, 2006 ▸) and used to spacially manipulate an X-ray beam (Blagov *et al.*, 2013 ▸) and control an acoustically tunable monochromator (Blagov *et al.*, 2015 ▸). Standing surface acoustic waves have been used for time and phase control of X-rays in stroboscopic diffraction experiments (Zolotoyabko & Quintana, 2002 ▸) and for fast modulation of the synchrotron X-ray beam intensity (Zolotoyabko & Quintana, 2004*a* ▸,*b* ▸). Recently, surface acoustic waves were also used in time-resolved coherent X-ray diffraction experiments (Reusch *et al.*, 2013 ▸; Nicolas *et al.*, 2014 ▸). This represents a first technical step in linking the experimental capabilities to perform time-resolved stroboscopic experiments at synchrotron sources with the imaging capability of coherent radiation.

In the present work we concentrate on surface acoustic waves (SAWs). SAWs propagate on the surface of a solid, parallel to it, and their amplitude shows an exponential decay in the bulk. SAWs modulate the surface of a crystal or a multilayer and can be used as a diffraction grating for X-ray radiation. The magnitude of the SAW phase velocity is 3000–5000 m s^−1^ (Erko *et al.*, 2008 ▸), which is five orders of magnitude slower than the speed of light. So, the SAWs can be described as a quasi-static grating, neglecting the Doppler effect influence on the light frequency. It has been shown that SAWs penetrate in the bulk up to the order of one SAW wavelength (Roshchupkin *et al.*, 2003 ▸). The diffraction of X-ray radiation on a superlattice in crystals or multilayers due to SAWs has been investigated in a number of publications (Dong *et al.*, 2010 ▸; Sauer *et al.*, 1998 ▸, 1999 ▸; Roshchupkin *et al.*, 1997 ▸, 1999 ▸; Tucoulou *et al.*, 2000 ▸). Different materials, such as LiNbO_3_, have been used as a carrier of the SAWs in different crystallographic geometries (Roshchupkin *et al.*, 1992 ▸, 1998 ▸; Tucoulou *et al.*, 2001 ▸). Appropriate theoretical models have been developed for the explanation of diffraction properties, especially the dependence of the diffracted intensity on the amplitude of the high-frequency signal applied to the device for the SAWs’ excitation (Punegov *et al.*, 2010 ▸; Schelokov *et al.*, 2004 ▸). The first example of such a device was reported by Tucoulou *et al.* (1997 ▸), who demonstrated for the first time the feasibility of a fast diffraction chopper for synchrotron radiation single-bunch pulses. The main advantage of this technique is its flexibility, that is the possibility to manipulate the parameters of the grating without moving any physical motor. It is in fact possible to control the wavelength and the amplitude of a dynamic SAW grating by changing the excitation frequency and the amplitude of the input high frequency. Unfortunately the time resolution of a chopper based on meridional diffraction on SAWs in this above-mentioned device was not enough to separate the BESSY II single pulse in hybrid operational mode, which is of the order of 100 ns. The large footprint size of the X-ray beam along the beam propagation direction and the low propagation velocity of SAWs limit the time resolution of the device.

Most of the mentioned research implements the standard meridional scattering geometry, where the X-ray beam is deflected in the scattering plane. The orientation of the optical element is tuned either to the Bragg angle to transmit the incoming beam or to the satellite angle to transmit the SAW modified diffraction satellite. Modification of the SAW amplitude modifies directly the intensity of the transmitted beam. Contrary to this, in the present paper we study SAWs in sagittal diffraction geometry, demonstrated for the first time by Roshchupkin, Irzhak *et al.* (2013 ▸), where the X-ray beam is diffracted normally to the scattering plane. This way both the original beam and the SAW satellites are transmitted simultaneously, and the intensity can be switched between them. Moreover, the time resolution of such a device benefits from this geometry, owing to the shorter propagation length needed for effective X-ray diffraction on SAWs. Recently, new materials such as langasite (LGS) crystals (La_3_Ga_5_SiO_14_) and langatate crystals (La_3_Ga
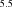
Ta
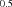
O_14_), developed for use in the microelectronics industry, were studied using SAW/X-ray methods in meridional geometry (Roshchupkin, Ortega *et al.*, 2013*a* ▸,*b* ▸; Roshchupkin, Irzhak *et al.*, 2013 ▸; Irzhak & Roshchupkin, 2014 ▸; Roshchupkin *et al.*, 2004 ▸, 2009 ▸). This new class of materials is of great interest because they exhibit a phase transition at high temperature (melt point) and high values of the piezoelectric constants. In this paper we also present the results of measurements in sagittal diffraction of a new class of piezoelectric material, LGS crystals, and compare them with the theoretically predicted intensities *versus* excitation voltage on a crystal.

## 2. Methods and material   

### 2.1. SAW device   

**Figure 1:**
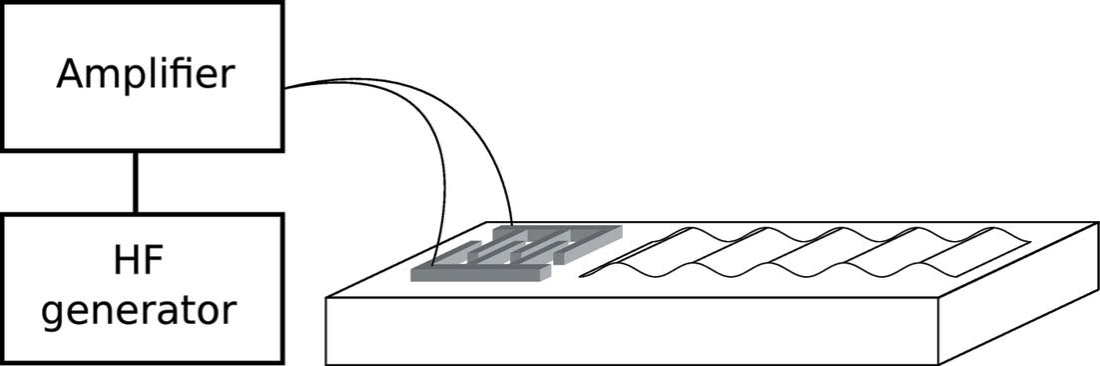
The SAW device. The IDT is connected to a high-frequency (HF) generator.

The excitation frequency (*f*) of SAWs depends on the speed (*v*_SAW_) of the SAWs on the exploited crystal and on the period (Λ) of the SAWs *via* the simple expression 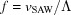. The deformation induced in the crystal to a first approximation can be written as 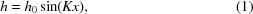
where 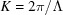 is the SAW wavenumber, 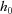 is the SAW amplitude on the crystal surface and *x* is the position on the crystal relative to the *x* axis, which is parallel to the propagation direction of the waves (Erko *et al.*, 2008 ▸). The substrate was an LGS crystal, of point group symmetry 32. The crystal lattice parameters are *a* = 8.170 Å and *c* = 5.095 Å. The crystal was polished to a roughness of approximately 5 Å. The crystal was grown along the [210] axis by the Czochralski technique at Fomos-Materials. For our sample the resonance frequency was 781 MHz, the SAW wavelength was 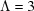
 µm and the propagation speed was 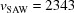
 m s^−1^. To excite SAWs, an interdigital transducer (IDT) made of aluminium was deposited on the surface of the LGS crystal. The structure of the IDT was written on the LGS substrate coated with (methyl methacrylate) resist by electron-beam lithography. The IDT converts the high-frequency electrical signal into acoustic oscillations of the crystal lattice that propagate along the crystal surface (Campbell, 1989 ▸) (Fig. 1 ▸). The amplitude of the SAWs depends linearly on the voltage supplied to the IDT, and it can easily be varied from zero to several ångströms.

### 2.2. Sagittal diffraction   

**Figure 2:**
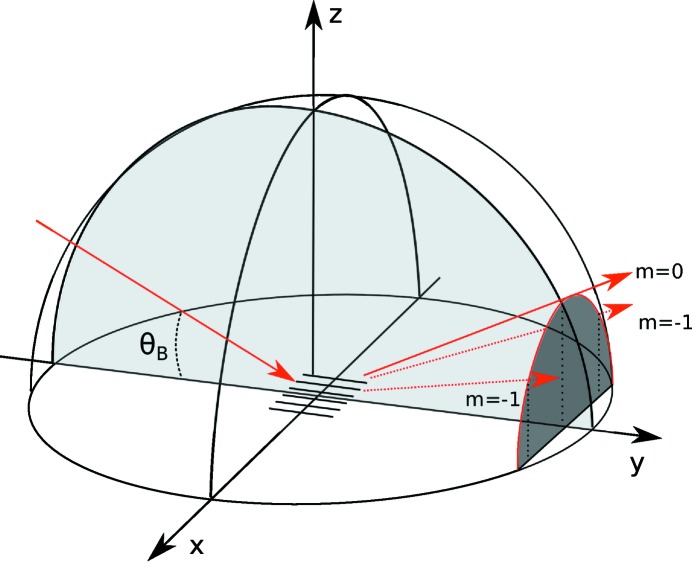
Sagittal diffraction. The grating created by SAWs lies in the *xy* plane, and the grooves of the grating are parallel to the scattering plane (light grey). The diffraction satellites propagate on the surface of a cone (cross section in dark grey). For simplicity, only the 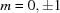 orders are represented in the diagram.

In sagittal diffraction geometry at the Bragg angle, the SAW wavefront is parallel to the scattering plane, as defined by the incoming and outgoing X-ray beams (Fig. 2 ▸). The diffraction takes place perpendicularly to the scattering plane. Therefore the Bragg diffracted and the SAW diffracted satellites appear simultaneously, and the diffraction satellites lie on the surface of a cone. The diffraction pattern are neither equally spaced nor positioned on a straight line. The angular separation of the *m*th order of diffraction can be written in the small-angle approximation as 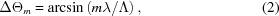
where λ is the wavelength of the incident radiation, Λ is the SAW wavelength and *m* is the diffraction order. Tucoulou *et al.* (2001 ▸) calculated the intensity of the diffraction satellites as
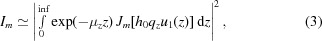
where 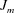 is the *m*th order Bessel function, 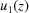 is the atomic displacement as a function of the depth in the crystal, μ*_z_* is the X-ray absorption coefficient and *q_z_* is the component of the momentum transfer vector along the *z* axis.

### 2.3. The X-ray SAW interaction   

Since the speed of light is five orders of magnitude higher than the speed of sound the acoustic deformation can be considered quasi-static. The interaction of X-rays with SAWs in the crystal can be divided into two distinct diffraction problems that can be treated separately. One can distinguish the Bragg diffraction from the diffraction due to SAWs that act as a grating. The Bragg angle depends only on the wavelength of the incident radiation λ and on the interplanar spacing *d* of the crystal *via* the Bragg law: 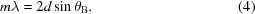
where 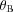 is the Bragg angle.

**Figure 3:**
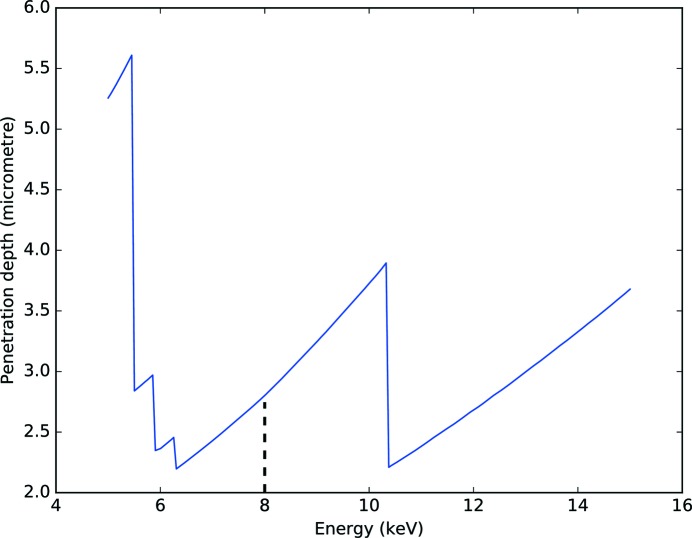
Penetration depth of X-rays in LGS for the 020 reflection.

The second diffraction problem is due to the lattice modulation introduced by SAWs. It can be treated as in the previous subsection, and it can be used to describe the diffraction process when SAWs are excited on the crystal surface. To study diffraction on SAWs it is convenient to select the case where the X-ray penetration depth is smaller than the SAW depth. In this way the X-rays interact only with the crystal planes that are distorted by SAWs. To describe the interaction of the X-rays with the crystal, if the depth of penetration exceeds the depth of the SAWs, the scattering from deep unmodulated crystal areas should be taken into account. Since we have a distorted crystalline lattice, the penetration depth is given by the absorption length and not by the extinction length. Thus we can use the kinematical approximation and calculate the X-ray penetration depth as a function of the X-ray energy: 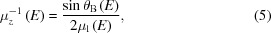
where 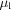 is the linear absorption coefficient. The dependence for the 020 reflection of the LGS crystal is shown in Fig. 3 ▸. The penetration depth at 8 keV is 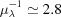
 µm, which is comparable to the depth of the SAWs, 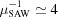
 µm.

Since the X-rays interact only with areas of the crystal modulated by SAWs, the intensities of the diffraction satellites can be calculated in the frame of the kinematical diffraction theory. The integral in equation (3) can be solved noticing that, as shown in Fig. 3 ▸, the modulation of the crystal lattice is constant in the portion of the sample in which the X-rays penetrate [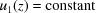]:
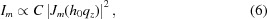
where *C* is a proportionality factor.

## 3. Experimental setup   

**Figure 4:**
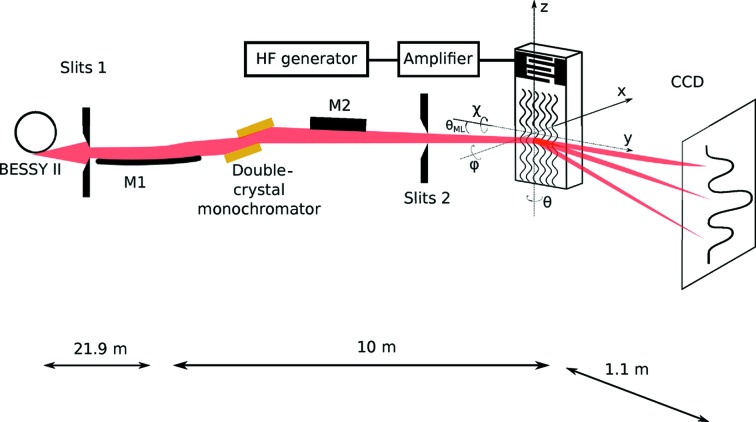
Experimental setup: M1 is a parabolic mirror that makes the beam parallel. The desired X-ray energy is then selected with a double-crystal Si(111) monochromator and the beam is focused onto the sample with a second mirror, M2. The beam size at the sample position can be varied using the second pair of slits.

Diffraction of X-ray radiation on an acoustically modulated LGS crystal was studied at the XPP-KMC 3 beamline (Reinhardt *et al.*, 2016 ▸) at the BESSY II synchrotron radiation facility (Fig. 4 ▸). The X-ray energy of *E* = 8 keV was selected by a double-crystal Si(111) monochromator, with an energy resolution 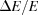 = 1/4000. The X-ray beam was cut with secondary slits of size 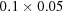
 mm (horizontal × vertical). The slits were closed vertically to 0.05 mm to increase the angular resolution. The intensity of the diffracted X-ray radiation was recorded with a CCD camera with pixel size of 6.5 µm (Proscan), sufficient to see the order separation with a CCD sample distance of 1.1 m. SAWs were excited using a high-frequency generator (Hameg, HM8134/5) and a wide-band radio frequency amplifier with 5 W power (AR, KAW1020). We used a Y-cut LGS crystal (reflection 020 and interplanar spacing 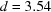
 Å) modulated by SAWs with wavelength 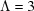
 µm. The Bragg incident angle was 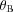 = 12.55°.

## 4. Experimental results   

**Figure 5:**
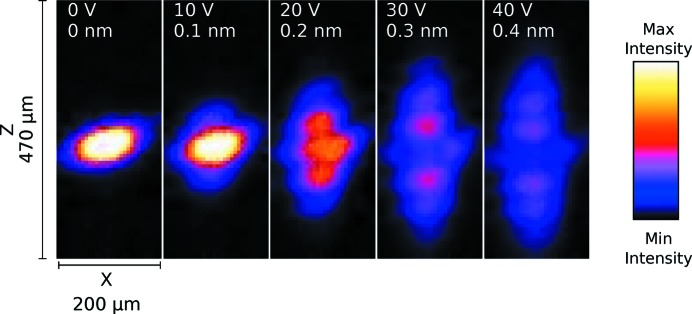
CCD camera images. The voltage supplied to the IDT was varied between 0 and 40 V, and consequentially the amplitude of the SAW changed. The orientation of the CCD camera is as shown in Fig. 4 ▸. The diffraction takes place along the *z* axis.

**Figure 6:**
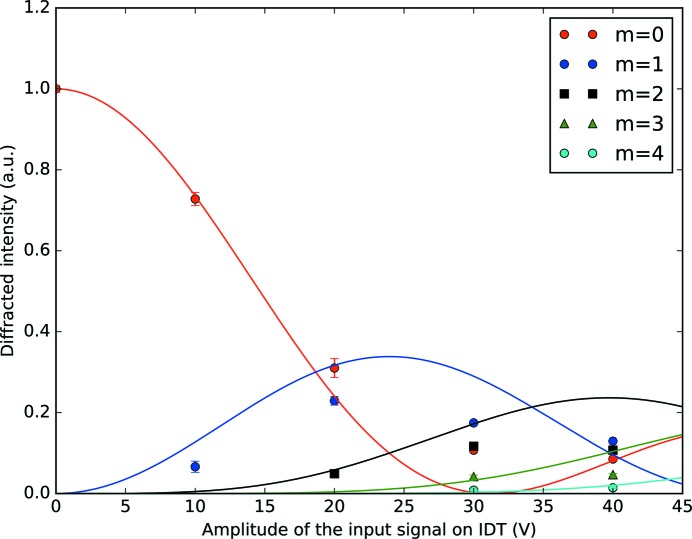
Intensities of the diffraction satellites (*m* = 0, 1, 2, 3, 4) *versus* the amplitude of the input signal supplied to the IDT. The circles, squares and triangles are the experimental data. The solid lines are the Bessel function squared, plotted as a visual reference.

The excitation of SAWs on the sample, a sinusoidal modulation of the crystal lattice, gives rise to diffraction satellites. The number and intensity of the satellites depend on the SAW amplitude. The measurements were taken at the 020 reflection. There is no need to rotate the crystal in sagittal geometry since the satellites appear together with the Bragg peak. We varied the amplitude of the SAWs by changing the voltage supplied to the IDT between 0 and 40 V in steps of 10 V. The relative angular separation between the diffraction satellites depends exclusively on the ratio between the SAWs and the radiation wavelength, as in equation (2). Fig. 5 ▸ shows the CCD camera images of X-ray Bragg diffraction on the SAW device. On increasing the amplitude of the SAWs, more diffraction satellites are visible and with higher intensity. The angular separation between the diffraction satellites is 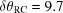
′′. Finally, we plot in Fig. 6 ▸ the normalized intensities of the diffraction satellites *versus* the amplitude of the input signal. We added to the plot the square of the Bessel function as a visual reference, according to the theoretical intensities as calculated with equation (6). For a qualitative analysis of the images during the experiment we used *ImageJ* (Abramoff *et al.*, 2004 ▸). For the quantitative analysis we wrote a Python script that automated the process. The intensity of the individual diffraction satellites was calculated by integration of selected regions.

## 5. Discussion   

**Figure 7:**
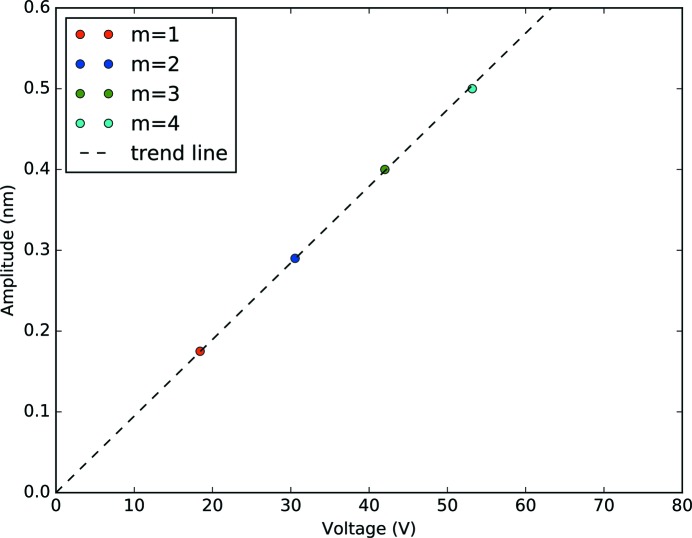
Amplitude of the SAWs plotted *versus* the voltage supplied to the IDT.

In Fig. 5 ▸, the Bragg peak (*m* = 0) and the diffraction satellites up to the third order are clearly visible, while the fourth order is revealed only after quantitative analysis. The Bragg peak and the satellites shift to lower *z* values when increasing the voltage. This is because the sample physically bends when the SAWs are excited. The measured angular separation between the diffraction satellites has 8% discrepancy with the theoretical value of 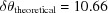
′′ calculated with equation (2). We simulated the intensity distribution among the diffraction orders using *GSolver* (http://www.gsolver.com/UserManual.pdf), a full vector solution of the diffraction grating problem for arbitrarily complex periodic grating structures that exploits the rigorous coupled wave analysis, a semi-analytical method to model the interaction of electromagnetic fields with physical objects. We compared the results shown in Fig. 6 ▸ with the simulations done in *GSolver*. We coupled the voltage at which the maxima occur in our experiment with the positions of the maxima as simulated in *GSolver*, which depend on the amplitude of the grating. We plot them one against each other in Fig. 7 ▸. This allows us to calculate the coupling constant *C* ≃ 0.1 nm V^−1^, which simply relates the voltage applied to the IDT with the amplitude of the generated SAWs. Using this constant it was possible to calculate the amplitude of the SAWs depending on the applied voltage (see Fig. 5 ▸). Note that this constant is not universal, but it has an intrinsic dependency on the setup. Many parameters may vary the *C* factor, such as the devices that are used to excite the SAWs (the high-frequency generator and the amplifier), as well as the cables and their length and the IDT design.

## 6. Conclusions and outlook   

The results of the theoretical and experimental investigations of X-ray Bragg diffraction in an LGS crystal excited by SAWs demonstrated the possibility to achieve effective diffraction of an X-ray beam in sagittal geometry. An appropriate theoretical model has been applied for calculation of the SAW amplitude and wavelength based on measurements of the electrical (amplitude of electrical signal) and diffraction (satellite intensity) parameters. The experimental results and theoretical predictions of kinematical theory are in a good agreement. This experiment demonstrates the feasibility of the implementation of a fast diffraction chopper for synchrotron radiation. The next step will be to use pulsed SAWs to isolate a single bunch in BESSY II.
